# Sec13 Regulates Expression of Specific Immune Factors Involved in Inflammation *In Vivo*

**DOI:** 10.1038/srep17655

**Published:** 2015-12-03

**Authors:** Thais G. Moreira, Liang Zhang, Lihi Shaulov, Amnon Harel, Sharon K. Kuss, Jessica Williams, John Shelton, Bandarigoda Somatilaka, Joachim Seemann, Jue Yang, Ramanavelan Sakthivel, Daniel R. Nussenzveig, Ana M. C. Faria, Beatriz M. A. Fontoura

**Affiliations:** 1 Department of Cell Biology, University of Texas Southwestern Medical Center, Dallas, TX 75390-9039, USA; 2 Department of Internal Medicine, University of Texas Southwestern Medical Center, Dallas, TX 75390-9039, USA; 3 Department of Pathology, University of Texas Southwestern Medical Center, Dallas, TX 75390-9039, USA; 4 Departamento de Bioquimica e Imunologia, Instituto de Ciencias Biologicas, Universidade Federal de Minas Gerais, Belo Horizonte, Brazil 31270-901; 5 Faculty of Medicine in the Galilee, Bar-Ilan University, Safed, Israel; 6 Veterans Affairs North Texas Health Care System: Dallas VA Medical Center, Dallas, TX 75216, USA

## Abstract

The Sec13 protein functions in various intracellular compartments including the nuclear pore complex, COPII-coated vesicles, and inside the nucleus as a transcription regulator. Here we developed a mouse model that expresses low levels of Sec13 (Sec13^H/−^) to assess its functions *in vivo,* as Sec13 knockout is lethal. These Sec13 mutant mice did not present gross defects in anatomy and physiology. However, the reduced levels of Sec13 *in vivo* yielded specific immunological defects. In particular, these Sec13 mutant mice showed low levels of MHC I and II expressed by macrophages, low levels of INF-γ and IL-6 expressed by stimulated T cells, and low frequencies of splenic IFN-γ+CD8+ T cells. In contrast, the levels of soluble and membrane-bound TGF-β as well as serum immunoglobulin production are high in these mice. Furthermore, frequencies of CD19+CD5-CD95+ and CD19+CD5-IL-4+ B cells were diminished in Sec13^H/−^ mice. Upon stimulation or immunization, some of the defects observed in the naïve mutant mice were compensated. However, TGF-β expression remained high suggesting that Sec13 is a negative modulator of TGF-β expression and of its immunosuppressive functions on certain immune cells. In sum, Sec13 regulates specific expression of immune factors with key functions in inflammation.

Nuclear import and export of molecules are vital cellular processes that occur through nuclear pore complexes (NPC). These pathways that mediate the translocation of molecules through the NPC are often usurped by pathogens to favor their replication^1^,^2^. As a countermeasure, the nuclear transport machinery responds to the abnormal challenge by regulating gene expression that favors the host. One example is the regulation of nucleoporin (nuclear pore complex proteins or Nups) levels by antiviral cytokines such as interferons^3^,^4^. In turn, nucleoporins can regulate gene expression by promoting nuclear export of mRNAs encoding antiviral factors and/or by regulating gene expression inside the nucleus where a pool of specific Nups are also found^5^,^6^,^7^,^8^. In the case of the nucleoporin Nup96, we have shown that it differentially regulates nuclear export of mRNAs encoding immune and cell cycle regulators^5^,^7^.

Additionally, several reports in plants have shown the involvement of nucleoporins in defense against pathogens. mos3-1, a homologue of Nup96 in plants, is required for defense against various pathogens, including parasite and bacteria^9^. mos3-1 regulates plant immunity and constitutive resistance via the de-regulated Toll interleukin 1 receptor/nucleotide-binding/leucine-rich repeat (TNL)-type R gene snc1^9^. Nup96 is a member of a complex of proteins termed the Nup107-160 complex. Other members of this complex, such as Nup160 and Seh1, also have functions in disease resistance in Arabidopsis^10^,^11^. Nup160 and Seh1 mediate constitutive resistance in the snc1 mutant and immunity via the TNL-type R genes as mos3-1, the homologue of Nup96^11^. This work has also shown that Nup160 and Seh1 have no roles in resistance mediated by coiled coil-type immune receptors, indicating specific functions for these nucleoporins in plant defense signaling. Furthermore, Nup160 mutant cells selectively reduced EDS1 levels, which is an important regulator of basal and TNL-triggered resistance. These results imply that Nup160 regulates proper expression of EDS1 and its functions in conditioned resistance pathways in plants, and that Nup160 may be required for EDS1 activities in autoimmunity^10^,^11^.

Another member of the Nup107-160 complex is Sec13, which is found at the nuclear pore complex^12^,^13^, inside the nucleus^12^,^14^, and is a constituent of the COPII vesicle coat in the cytoplasm^15^,^16^. We have previously shown that Nup96 interacts with Sec13^12^ and that Nup96 has specific roles in immunity related to interferon expression and function in mice^7^. Nup96^+/−^ mice have impaired IFNα- and γ-mediated induction of MHC I and IFNγ-mediated induction of MHC II, ICAM-1, and other proteins. The Nup96^+/−^ mice also presented a diminished frequency of CD3+TCRαβ+ and CD4+ T cells, which depends on MHC function. These defects resulted in abnormal antigen presentation and T cell proliferation during immunization and susceptibility to viral infection^7^.

Here, we investigated the function of Sec13 *in vivo*. We have developed a new mouse model that expresses low levels of Sec13. While many immunological and cellular biological parameters were normal in these Sec13 hypomorphic mice (Sec13^H/−^), they presented specific immunological defects related to interferon gamma (IFNγ), IL-6, TGF-β expression, and immunoglobulin production. Among these phenotypes, the defect in TGF-β expression is the one that persisted upon immunization. TGF-β plays a critical role in restraining pathogenic Th1 responses *in vivo* by inhibiting proinflammatory responses including macrophage activation, T cell proliferation, and IFNγ expression^17^,^18^. A direct connection between TGF-β and the development of T cells with regulatory properties (Tregs) has been reported. TGF-β has been shown to induce Foxp3, a transcription factor that is a master regulator of Tregs in naïve T cells and to be expressed coupled to a latency-associated peptide (LAP) on the surface of a subset of Tregs. LAP has been identified as a marker that distinguishes activated Tregs, which mediate suppression via TGF-β^19^. Here, Sec13^H/−^ mice showed elevated frequencies of LAP+ regulatory T (Treg) cells as well as decreased expression of IFNγ and of the pro-inflammatory cytokine IL-6. These results were accompanied by low protein levels of interferon-regulated genes such as MHC class I and MHC class II. Presentation of antigenic peptides in the pockets of MHC class I and II is an essential step for the generation and activation of mature CD8+ and CD4+ T lymphocytes in the peripheral lymphoid tissues^20^. Moreover, Sec13^H/−^ mice presented high levels of serum non-specific immunoglobulins. Overall, these studies reveal specific immunosuppressive alterations in Sec13^H/−^ mice that are known to impact inflammation.

## Results

### Mice Expressing Low Levels of Sec13 Did Not Present Pleiotropic Defects

**Figure 1:**
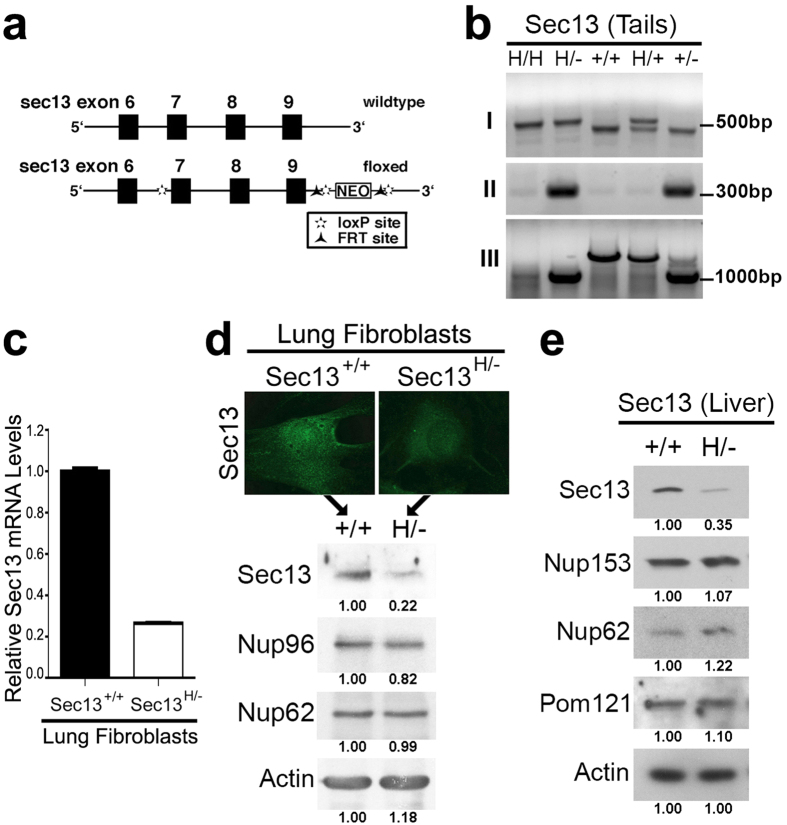
Targeted Disruption of the Sec13 Gene in Mice Decreased Sec13 mRNA and protein Levels. (**a**) Schematic representation of part of the Sec13 wild-type gene and the mutant allele. Exons 7, 8 and 9 were deleted using the Cre recombinase. (**b**) Genotyping was performed with primer sets that detect: (I) the wild-type allele and the hypomorphic allele containing the full construct including exons 7, 8, and 9, NEO cassette, FRT and loxP sites; (II) the allele deleted of exons 7, 8, and 9; (III) the wild-type allele and the allele deleted of exons 7, 8, and 9. (**c**) Sec13 mRNA levels were measured in wild-type and Sec13^H/−^ mouse lung fibroblasts by real time-RT PCR. GAPDH and actin were used as controls. (**d**,**e**) Sec13^+/+^ and Sec13^H/−^ lung fibroblasts were subjected to immunofluorescence microscopy with anti-Sec13 antibodies (**d**) and immunoblot analysis with antibodies against the depicted proteins (**e**). (**f**) Immunoblot analysis of liver extracts from Sec13^+/+^ and Sec13^H/−^ cells using antibodies against the depicted proteins. Immunoblots were quantified by ImageJ software.

Since Nup96 mice presented specific immune defects and Sec13 interacts with Nup96, we set out to investigate the function of Sec13 in mice. A 13kb region containing most of the *sec13* gene was used to construct the targeting vector. This region was first subcloned from a positively identified BAC clone (B6) and designed so that the short homology arm (SA) extends 1.4 kb from the 3′ of exon 9. The long homology arm (LA) starts 5′ of exon 7 and is ~8.7 kb long. The Neo cassette was inserted downstream of the 3′ end of exon 9 and is flanked by two loxP sites. A single loxP site was also inserted upstream the 5′ end of exon 7. The target region is ~2.8 kb and includes exon 7, 8 and 9. After crossing with mice expressing the Cre recombinase, a mutated allele of *sec13* lacking exons 7, 8 and 9 was generated (Fig. 1a,b). Sec13^−/−^ mouse was not viable indicating that the truncated Sec13 was likely degraded. However, we were able to obtain mice expressing low levels of full-length Sec13 protein, Sec13^H/−^ mice. These mice lacked exons 7, 8 and 9 in one allele, as above, and the second allele encodes the same construct with loxP sites but no deletions (Fig. 1b). The presence of the construct in this allele, without deletion, lowered full-length Sec13 mRNA and protein levels (Fig. 1c–e). This is likely because the neo gene creates a cryptic neo exon, which can be included or excluded during splicing. This effect generates a certain amount of truncated protein that is likely degraded but also yields the full-length transcript. Thus, we detected low levels of full-length Sec13 in the Sec13^H/−^ mice as compared to Sec13^+/+^ mice. While we show that full-length Sec13 protein levels are low in Sec13^H/−^ mice, this abnormality did not significantly impact the levels of other tested nucleoporins (Fig. 1d,e). Nup96 levels appear slightly reduced in Fig. 1d (18%) but the average between three independent experiments shows a 4% decrease, which is not significantly different than wild-type levels.

**Figure 2:**
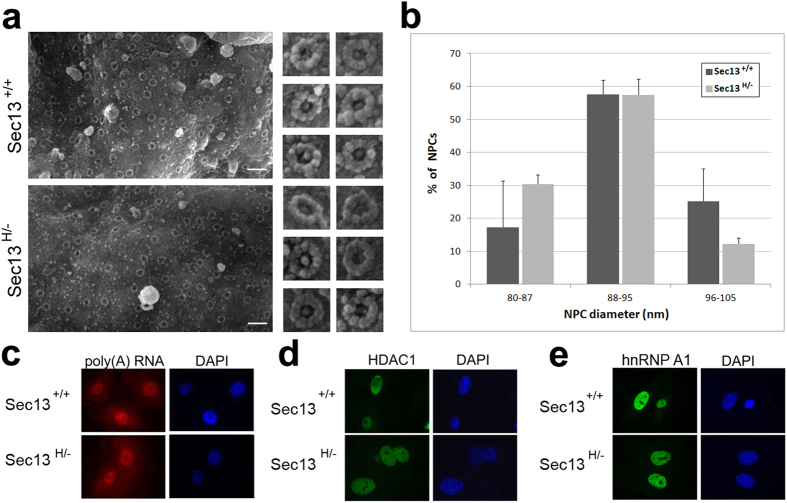
Nuclear Pore Complex Structure and Bulk Nucleo-Cytoplasmic Trafficking Are Not Significantly Altered in Sec13^H/−^ Cells. (**a**) Sec13^+/+^ and Sec13^H/−^ lung fibroblasts were subjected to hypotonic treatment in order to expose nuclei, and prepared for FESEM imaging. Two large representative areas of nuclear envelopes are shown on the left along with a gallery of individual NPCs from each cell type. (**b**) Histogram shows a quantitative summary of the apparent external diameter measured only in NPCs embedded in flat membrane areas and viewed from the top. No significant architectural differences were detected between NPCs from Sec13^+/+^ or Sec13^H/−^ cells. (**c**) Sec13^+/+^ and Sec13^H/−^ lung fibroblasts were subjected to oligo-dT *in situ* hybridization to determine the intracellular distribution of poly(A) RNA. (**d**,**e**) Sec13^+/+^ and Sec13^H/−^ lung fibroblasts were subjected to immunofluorescence microscopy with anti-HDAC1 antibody (**d**) or anti-hnRNP A1 antibody (**e**), respectively.

We then investigated the structure of the nuclear pore complex (NPC) in Sec13^H/−^ cells. To assess the reduction of Sec13 protein levels on NPC structure, we used field emission scanning electron microscopy (FESEM), which provides high-resolution three-dimensional surface images of the cytoplasmic side of the nuclear envelope. As shown in Fig. 2a,b, FESEM imaging of exposed nuclei from lung fibroblasts revealed large expanses of nuclear envelopes with NPCs embedded in intact membranes. Comparison of Sec13^+/+^ and Sec13^H/−^ cells showed no significant differences in the distribution of NPCs or in their visible architectural features. Thus, low levels of Sec13 in Sec13^H/−^ mice do not appear to cause any gross architectural changes in the NPC, as visualized from the cytoplasmic side of the nuclear envelope.

To determine whether Sec13^H/−^ mice presented defects in nucleo-cytoplasmic trafficking, transport of mRNA and proteins were assessed. Oligo-dT *in situ* hybridization was performed to investigate the intracellular distribution of poly(A) RNA and showed no significant changes between the Sec13^H/−^ mice and their wild-type counterparts (Fig. 2c). These lung fibroblasts have very large cytoplasm with poly(A) RNA distributed throughout the whole area, giving the false impression that there is considerably more poly(A) RNA in the nucleus. In addition, we performed immunofluorescence staining to determine the intracellular distribution of HDAC1 and hnRNP A1, which are imported into the nucleus via the Karyopherin α/β1 and β2 transport pathways, respectively. In both cases, we have observed no differences between the Sec13^H/−^ and Sec13^+/+^ cells (Fig. 2d,e), indicating that bulk transport through the nuclear pore complex is not significantly altered. This is not surprising as Sec13^H/−^ mice are viable and do not present gross abnormalities.

**Figure 3:**
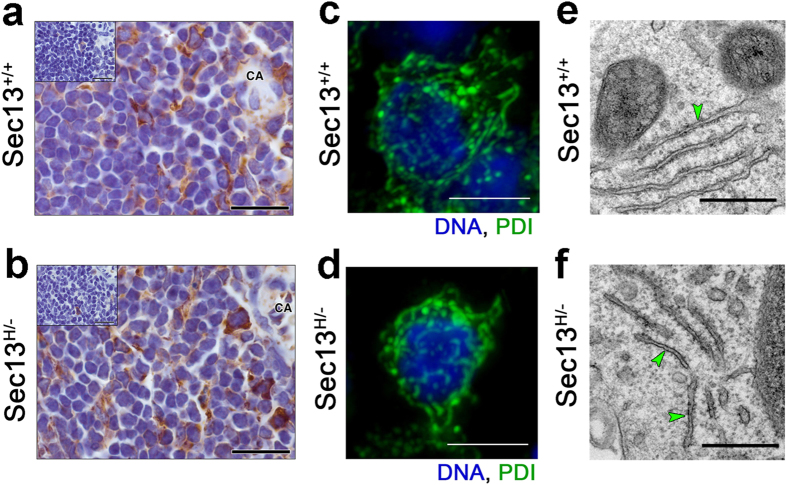
Reduced Levels of Sec13 Protein Do Not Alter ER Morphology. (**a**,**b**) Immunohistochemistry for protein disulfide-isomerase (PDI). High magnification bright-field microscopy of PDI immunoperoxidase localization in the spleen of Sec13^+/+^ and Sec13^H/−^ mice. Boundaries of endoplasmic reticulum are defined by PDI staining (brown) in the spleen of wild-type and mutant mice. The cytoplasm of lymphocytes adjacent to the splenic central arteriole also stain positive for PDI. Hematoxylin counter stain is blue/purple and inset micrographs show absence of staining in matching-anatomy from adjacent sections not subjected to primary antibody. CA, central arteriole; bars, 20 μm. (**c**,**d**) Lymphocytes from spleen of Sec13^+/+^ and Sec13^H/−^ mice were subjected to immunofluorescence with anti-PI antibody followed by Deconvolution Microscopy. Bars, 5 μm. (**e**,**f**) Spleen from Sec13^+/+^ and Sec13^H/−^ mice were processed for thin sectioning and observed by Electron Microscopy. ER is marked by green arrowheads. Bars, 0.5 μm.

**Figure 4:**
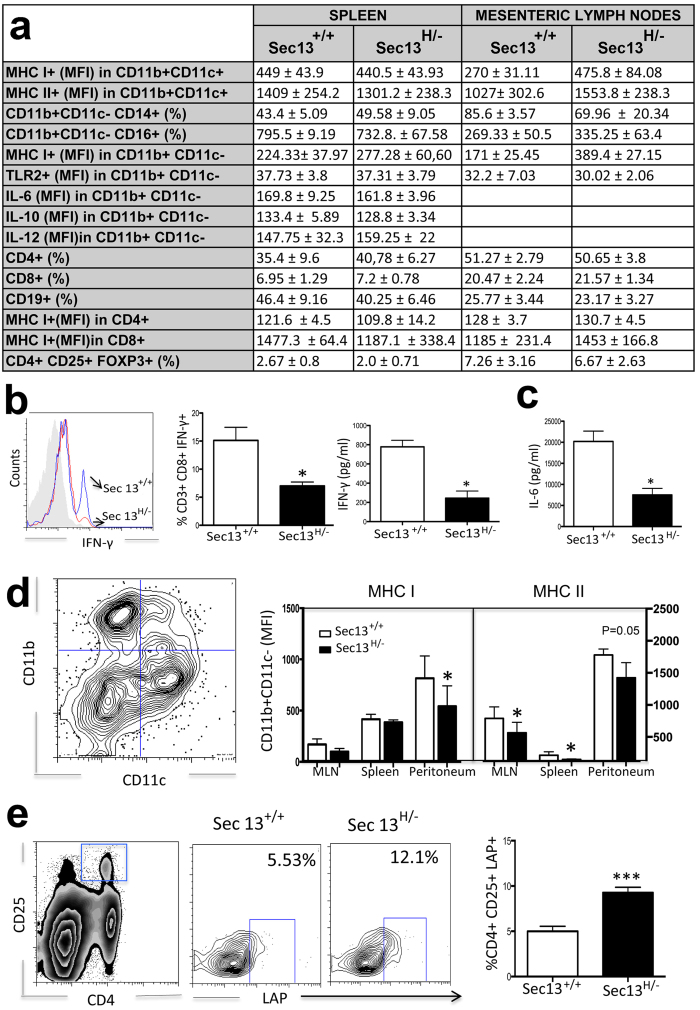
Sec13^H/−^ Mice Present Specific Immunological Alterations. (**a**) Analysis of immunological cell populations in Sec13^+/+^ and Sec13^H/−^ mice. Cell subsets isolated from spleen and mesenteric lymph nodes (MLN) were analyzed by flow cytometry using CD4+ and CD8+ as phenotypic markers for T lymphocytes, CD19+ for B lymphocytes, and CD11b+CD11c- for macrophages. Results are the mean ± SD (n = 8). Intracellular cytokine production (IL-6, IL-10, and IL-12) was measured by flow cytometry in cultured cells in the presence of ConA and GolgiStop®. (**b**–**e**) Splenic cells from Sec13^+/+^ and Sec13^H/−^ mice were subjected to flow cytometry to analyze frequencies of specific populations and expression of molecules. Cells were harvested and cultured for 48h in the presence of Con-A. Intracellular cytokine production was measured by flow cytometry in cultured cells in the presence of ConA and GolgiStop®. Cells were also stained with FITC-labeled anti-CD8, PercP-labeled anti-CD3 and PE-labeled anti-IFN-γ. Bar graphs are shown as mean ± SEM. (**b**,**c**) Cytokine production by splenic cells was measured in the supernatant by sandwich ELISA. Bar graphs are shown as mean ± SEM. (**d**) MHC I and MHC II expression was measured in CD11b+ CD11- cells isolated from mesenteric lymph nodes, spleen, and peritoneum. Graphs are representative of 6 mice/group. (**e**) Cells were stained with FITC-labeled anti-CD4, PercP-labeled anti-CD25, and PE-labeled anti-LAP. CD4+ cells and CD25+ were gated. LAP+ cells were gated within CD4+ CD25+ cells. Plots are representative of the mean of 3 mice/group. All data are representative of three independent experiments. Bar graphs are shown as mean ± SEM. Student T test was applied. *p < 0.05; ***p < 0.0005.

**Figure 5:**
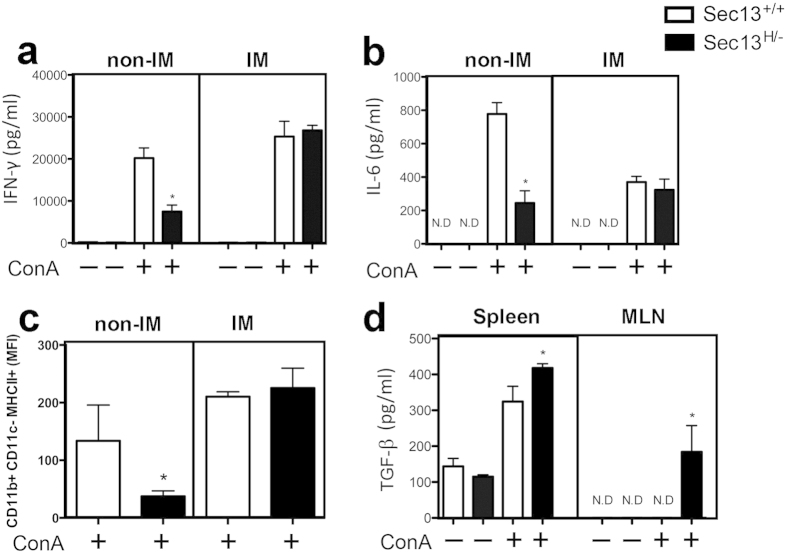
Immunization Counteracts Some of Sec13^H/−^ Immunological Defects. Sec 13^+/+^ and Sec13^H/−^ mice were either non-immunized or immunized with ovalbumin adsorbed in a Th1 adjuvant, *Mycobacterium tuberculosis*-containing Complete Freund’s Adjuvant. Spleen cells from non-immunized or immunized Sec13^+/+^ and Sec13^H/−^ mice were stimulated *in vitro* with ConA. MHC expression and cytokine production were then analyzed after 48 hours. Results are the mean ± SD (n = 8). All data are representative of three independent experiments. NI, non-immunized; IM, immunized.

**Figure 6:**
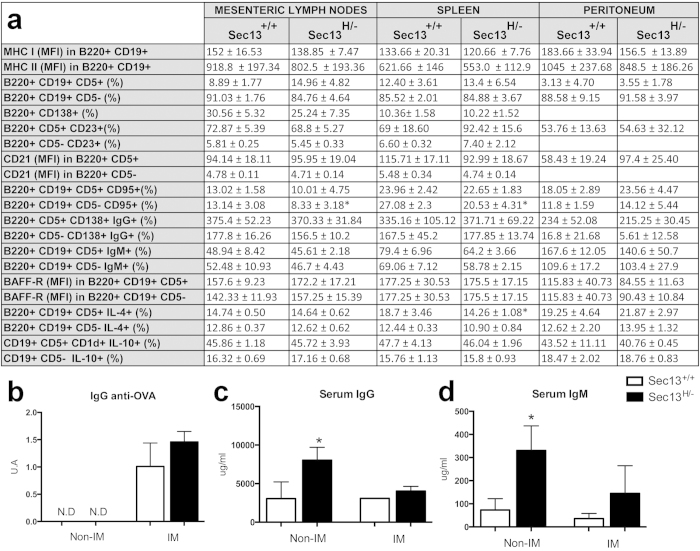
Sec13^H/−^ mice present hypergammaglobulinemia that is reversed upon Immunization. (**a**) Analysis of B cell phenotype in Sec13^+/+^ and Sec13^H/−^ mice. Cell subsets isolated from spleen and mesenteric lymph nodes (MLN) were analyzed by flow cytometry using B220+ CD19+ as phenotypic markers for B lymphocytes. B1 cells were identified as either B220+CD19+CD5+ or CD19+CD5+CD1d+. The activation state of B cells was studied using CD23, CD138 as markers; B cell survival was analyzed using BAFF-R and CD95 as markers. Results are the mean +/− SD (n = 8). Data are representative of three independent experiments. Student T test was applied. *Statistically different p < 0.05. (**b**–**d**) Immunoglobulin levels were determined in sera of Sec13^+/+^ and Sec13^H/−^ mice by ELISA. Results are the mean +/− SD (n = 8). Data are representative of three independent experiments.

We then investigated whether low levels of Sec13 altered ER structure. The PDI (protein disulfide isomerase) marker was used in immunohistochemistry of spleen (Fig. 3a,b) and liver (Supplementary Figure 1a) sections to assess gross defects in ER structure in lymphocytes and hepatocytes from Sec13^+/+^ and Sec13^H/−^ mice. No gross ER defects were observed at the tissue level in Sec13^H/−^ mice. In addition, ER images of lymphocytes were obtained by immunofluorescence with anti-PDI antibody followed by Deconvolution microscopy (Fig. 3c,d) and showed no differences between wild-type and mutant Sec13 mice. Furthermore, lymphocyte areas from spleen of Sec13^+/+^ and Sec13^H/−^ mice were subjected to thin sectioning followed by Electron Microscopy (TEM) and, again, the ER morphology was not altered in Sec13^H/−^ mice (Fig. 3e,f). We have also analyzed the secretory function of immune cells from Sec13^+/+^ and Sec13^H/−^ mice as well as the levels of certain factors secreted by the liver. Overall there is no bulk defect in secretion but the levels of certain specific immune factors that traffic through the secretory pathway are abnormal in Sec13^H/−^ mice. These data are discussed below (Figs 4, 5, 6). The levels of key factors secreted by the liver were not statistically different between the wild-type and mutant mice, falling within the expected variability in mice (Supplementary Figure 1b). Moreover, ER exit sites and Golgi structure were assessed by staining mutant and wild-type Sec13 cells with antibodies against Sec31 and p115, respectively. As shown in Supplementary Figure 2a, ER exit sites or Golgi morphology remain intact in Sec13^H/−^ cells. We also show that Sec31 level is reduced in Sec13^H/−^ mice as compared to wild-type mice (Supplementary Figure 2b). However, these decreased levels of Sec31 is not sufficient to affect the pool of Sec31 localized at the ER exit sites (Supplementary Figure 2a), suggesting that cytoplasmic Sec31 is the pool reduced and is not rate limiting for Sec31 recruitment to ER exit sites. This is corroborated by no changes in bulk secretory functions observed in Sec13^H/−^ mice.

### Low Levels of Sec13 in Mice Result in Specific Immune Defects

Since Sec13 interacts with Nup96 and the latter regulates specific immune functions, we analyzed several aspects of immunity in the Sec13^H/−^ mice^5^,^7^,^9^. Expression of a panel of immunologically relevant molecules was analyzed by flow cytometry in isolated cells from spleen and mesenteric lymph nodes. Various immunological cell populations and functions were normal in Sec13 hypomorphic mice (Fig. 4a). Sec13^H/−^ mice presented normal frequency of T and B cells and granulocytes (Fig. 4a). Production of certain anti-inflammatory and pro-inflammatory intracellular cytokines (IL-6, IL-10, and IL-12) was also normal in macrophages (Fig. 4a). In addition, expression of CD14 and CD16 in these cells was not altered, suggesting normal leucocyte differentiation and activation (Fig. 4a). On the other hand, splenic T cells from Sec13^H/−^ express low levels of intracellular IFN-γ (Fig. 4b). Furthermore, IFN-γ (Fig. 4b) and IL-6 (Fig. 4c) secreted levels were low in cell culture supernatants from Sec13^H/−^ splenic cells that were stimulated for 48h with the mitogen concanavalin A (Fig. 4b,c). The reduction of IFN-γ levels in Sec13^H/−^ mice was followed by a slight but significant decrease in expression of interferon regulated genes such as MHC I and MHC II in macrophages, from spleen and mesenteric lymph nodes (Fig. 4d). We did not observe any difference on the frequency of regulatory CD4+ T cell population expressing the transcription factor Foxp3 (Fig. 4a). However, we detected high frequencies of CD4+ T lymphocytes expressing the membrane form of TGF-β1 coupled to LAP in Sec13^H/−^ mice (Fig. 4e).

As the interaction of MHC molecules and their peptide content with TCRs in T cells is critical to activate immune responses, we tested whether the reduced levels of MHC expression would affect cytokine production in splenic cells. Sec13^+/+^ and Sec13^H/−^ mice were immunized with ovalbumin and a potent Th1 adjuvant, *Mycobacterium tuberculosis*-containing CFA. We have chosen this type of stimulation because it induces a Th1 response with production of IFN-γ. Spleen cells from immunized Sec13^+/+^ and Sec13^H/−^ mice were stimulated *in vitro* with concanavalin A. MHC expression and cytokine production were then analyzed after 48 hours. Surprisingly, we observed that after immunization IFN-γ, IL-6, and MHC II levels became similar between Sec13^+/+^ and Sec13^H/−^ mice while secreted TGF-β was still higher in Sec13^H/−^ mice than in Sec13^+/+^ mice (Fig. 5). These results suggest that inflammatory challenge induces recovery of some but not all immune functions in Sec13^H/−^ mice. The mRNA levels of these cytokines were also measured by real time RT-PCR (Supplementary Figure 3) and only TGF-β showed a slight increase in mRNA level at 5 h post-concanavalin A treatment, indicating that up-regulation of both mRNA and protein contributed to the higher levels of secreted TGF-β in Sec13^H/−^ mice than in Sec13^+/+^ mice, especially in the mesenteric lymph nodes.

TGF-β regulates B cell development and function^21^. Therefore, B cells were also analyzed using their functional markers and by assessing immunoglobulin levels in the serum (Fig. 6). We found no significant difference in the frequencies of B1 (B220+CD19+CD5+) and B2 (B220+CD19+CD5–) cells in the lymphoid organs examined (Fig. 6a). Using the expression of FcεRII (CD23) as marker of naïve B cells and CD21 and CD138+ as markers of activated and Ig-secreting B cells^22^ respectively, we did not observe differences between Sec13^H/−^ and wild type mice (Fig. 6a). IL-4-producing B effector 2 cells^23^ and IL-10-producing regulatory B cells^24^ analyzed were also present at the same frequencies in both groups (Fig. 6a). On the other hand, frequencies of B1 and B2 cells expressing the Fas-receptor (CD95) were lower in Sec13^H/−^ mice than in Sec13^+/+^ mice (Fig. 6a). Expression of molecules that mediate B cell function and activation were also evaluated. Neither expression of MHC II nor MHC I in B cells were altered in Sec13^H/−^ mice (Fig. 6a). In addition, no differences were observed in the expression of receptors for the B cell activating factor (BAFF) in the surface of B1 and B2 cells (Fig. 6a). Interestingly, Sec13^H/−^ mice had higher levels of serum Ig, IgG, and IgM than their wild type counterparts (Fig. 6c,d). This increase in Ig, IgM and IgG production was reverted by immunization with the protein antigen ovalbumin in complete Freund’s adjuvant (CFA) concomitantly with the increase in serum specific anti-OVA Ig production (Fig. 6b–d).

We have also tested interferon-β production by transfecting low and high molecular weight forms of poly (I:C) into fibroblasts of Sec13^+/+^ and Sec13^H/−^ mice. These two forms of poly (I:C) are synthetic RNAs that mimic the effect of viral infection by activating the RIG-I and MDA5 pathways leading to type I IFN expression^25^. Both forms of poly (I:C) similarly induced IFN-β expression in Sec13^+/+^ and Sec13^H/−^ cells (Supplementary Figure 4), indicating no apparent alterations in these pathways. In sum, Sec13^H/−^ mice revealed a specific immunosuppressive phenotype.

## Discussion

In this study, we investigated the role of Sec13 *in vivo* using a mouse model in which Sec13 is expressed at low levels without affecting its functions in bulk nucleo-cytoplasmic trafficking and in the secretory pathway. We showed that Sec13^H/−^ mice displayed a few similarities with Nup96^+/−^ mice^7^. Both Sec13^H/−^ and Nup96^+/−^ mice presented selective defects in the expression of key IFNγ-regulated genes such as MHC expression. Both MHC class I and class II are regulated by cytokines including IFN-γ^26^,^27^. Sec13^H/−^ mice showed low levels of INF-γ and IL-6 produced by stimulated T cells as well as low frequencies of splenic IFN-γ+CD8+ T cells. As expected, Sec13^H/−^ mice have decreased levels of MHC I and MHC II in macrophages. In the Nup96^+/−^ mice, MHC expression was also down-regulated but IFN-γ level was high^7^. The increased levels of IFN-γ in the Nup96^+/−^ mice was likely a compensatory mechanism for the reduced expression of interferon-induced genes^7^. At the same time that Sec13^H/−^ mice have low levels of IFN-γ, showing no compensation for the decrease in the expression of interferon-regulated genes as in the case of the Nup96^+/−^ mice, Sec13^H/−^ mice present high frequency of regulatory T (Treg) cells producing high levels of the surface and secreted forms of TGF-β1. Among the important immunosuppressive activities of TGF-β1 are down-regulation of T-bet, an IFN-γ-inducible transcription factor that promotes Th1 differentiation^28^, and inhibition of IFN-γ expression^17^. These findings suggest a potential defect in the crosstalk between the IFN-γ and TGF-β1 regulatory loop in the presence of low Sec13 levels. These low levels of Sec13 did not affect many immunological functions, including IFN-β levels and various immunological cell populations and factors, indicating specific defects of immunity.

An interesting feature of the Sec13^H/−^ mice is the reversion of some of these phenotypes after immunization with antigen in the presence of Freund’s adjuvant (CFA). While IFN-γ and IL-6 production by spleen cells as well as MHCII expression by macrophages (CD11b+ cells) were restored in Sec13^H/−^ mice, secretion of TGF-β by Con-A-stimulated cells from spleen and MLN was increased upon immunization. CFA is a potent stimulator of Th1 responses via secretion of IL-12, and this cytokine negatively regulates differentiation programs induced by TGF-β such as Foxp3 and RORγt expression^29^. IFN-γ itself suppresses TGF-β production and signaling through up-regulation of the inhibitory Smad 7^30^,^31^ and via direct interaction of YB-1 with Smad3^32^. Moreover, Th1-related cytokines are dominant over TGF-β stimulation being able to skew already ongoing TFG-β-dependent responses^29^. Indeed, we observed that upon immunization IFNγ production was restored in Sec13^H/−^ mice as well as their related responses such as MHC II expression while high levels of TGF-β were concomitantly produced by spleen and MLN cells (Fig. 5). Therefore, the parallel and divergent alterations in the reciprocally regulated IFNγ and TGF-β responses suggest that these pathways are independently influenced by Sec13.

In addition, other potentially inflammatory immune factors are affected by lowering the levels of Sec13 expression. IL-6 was down-regulated in Sec13^H/−^ mice. This cytokine is known as a potent pro-inflammatory mediator secreted by several cell types, such as macrophages, dendritic cells, adipocytes, and is involved in a myriad of acute and chronic inflammatory diseases and processes^33^. Therefore, the net result of reducing the levels of Sec13 *in vivo* seems to be a reduced pro-inflammatory profile of the animal.

Regarding B cell function, the most remarkable difference found in Sec13^H/−^ mice was the increased serum levels of immunoglobulins of both IgM and IgG isotypes. In spite of that, there was no alteration in frequencies from either B1 or B2 cell subsets expressing markers of activation. Serum levels of BAFF (data not shown) as well as frequencies of BAFFR+ B cells were normal. However, the increase in non-specific immunoglobulin production correlated with low frequencies of B1 and B2 cells expressing the Fas-receptor (CD95), a factor that induces apoptosis when bound by Fas ligand. Previous *in vivo* data indicate that the defect in Fas function results in uncontrolled autoantibody production, autoimmunity, and increased risk of B cell lymphomas, revealing that the Fas/FasL balance must be very accurately regulated during humoral immune response^34^. Fas plays a role in the elimination of non-specific and autoreactive B cells in the germinal center. Antigen-specific survival such as B cell receptor (BCR), or MHC II signal, or coreceptors (CD19) cooperating with BCR inhibits the formation of the death inducing signaling complex^35^,^36^,^37^,^38^. Indeed, hypergammaglobulinemia has been reported in autoimmune diseases such as lupus erythematosus^39^, Sjögren’s syndrome^40^, rheumatoid arthritis^41^, as well as in the acquired immunodeficiency caused by HIV infection^42^,^43^. The specificity of the antibodies found in patients with hypergammaglobulinemia is still a matter of debate. Pathogenic autoantibodies detected in autoimmune diseases are predominantly IgG isotypes, reflecting the generation and activation of an autoimmune memory B cell repertoire. In HIV patients, however, they are not directed towards autoantigens as well as microbiota antigens^44^. In our study, we have shown that immunization with an antigen (ovalbumin) in the presence of CFA led to recovery of normal levels of polyclonal serum antibodies (IgM and IgG) followed by an increase in the production of specific serum antibodies to OVA. On one hand, polyclonal immunoglobulin production in the presence of high frequencies of TGF-β-positive T cells and unaltered frequencies of activated B cells resemble the immunosuppressive pattern seen in an immunodeficiency scenario. On the other hand, the recovery of most of B and T cell functions upon immunization showed that low levels of Sec13 expression were sufficient to allow recovery of a normal phenotype when the immune system was robustly stimulated.

The only alteration that immunization was not able to overrule was the TGF-β expression suggesting that Sec13 represents a key element for TGF-β production. We demonstrated an increased frequency of the CD4+ activated (CD25+) T cells that express TGF-β in their surface (LAP) in Sec13^H/−^ mice. These cells can be precursors of CD4+ T cells that secrete TGF-β (Th3 cells)^19^,^45^,^46^. Thus, production of the membrane versus the secreted form of TGF-β represents distinct stages of differentiation of TGF-β-producing CD4+ T cells. We then showed that the levels of secreted TGF-β are elevated in Sec13^H/−^ mice when T cells are stimulated with ConA. In this case, stimulation by ConA was required to induce TGF-β-producing T cells *in vitro* and to reveal the effect of Sec13 on the production of the secreted form of TGF-β.

While Sec13 has functions in bulk nucleocytoplasmic trafficking^12^,^13^ and in the secretory pathway^16^, Sec13 regulates transcription of a subset of genes^12^,^14^. It is possible that the specific immune defects observed in the Sec13^H/−^ mice are related to one or more functions of Sec13 in these different compartments, which could be the topic of future studies. Since some, but not all, of the defects observed in the Sec13^H/−^ mice are similar to the Nup96^+/−^ mice, including down-regulation of MHC expression^7^, these results suggest a role for these proteins in the same immune pathway. Similar to a potential role of Sec13 in autoimmunity, Nup160, which is in the same complex with Nup96 and Sec13, regulates EDS1 expression as well as its functions in conditioned resistance pathways and autoimmunity^10^,^11^. In sum, we have uncovered novel and specific functions of the Sec13 protein in immunity.

## Material and Methods

### Generation of Sec13^H/−^ mice

A 13kb region used to construct the targeting vector was first subcloned from a positively identified BAC clone (B6). The region was designed such that the short homology arm (SA) extends 1.4 kb from 3′ exon 9. The long homology arm (LA) starts at 5′ of exon 7 and is ~8.7 kb long. The loxP flanked the Neo cassette and is inserted at the 3′ side of exon 9, and the single loxP site is inserted at the 5′ side of exon 7. The target region is ~2.8 kb and includes exon 7, 8 and 9. The targeting vector was confirmed by restriction analysis after each modification step. P6 and T7 primers anneal to the vector sequence and read into the 5′ and 3′ ends of the BAC sub clone. N1 and N7 primers anneal to the 5′ and 3′ ends of the Neo cassette and sequence the SA and LA respectively. PCR primers used for sequencing included: Primer P6 5′-ATTTAGGTGACACTATAGAACTC-3′; Primer T7 5′-ATTATGCTGAGTGATATCCCTCT-3′; Primer N1 5′-TGCGAGGCCAGAGGCCAGTTGTGTAGC-3′; Primer N7 5′-ATGTGTCAGTTTCATAGCCTGAAG-3′.

Backbone Vector Information: The BAC was sub cloned into a ~2.4kb backbone vector containing an ampicillin selection cassette for retransformation of the construct prior to electroporation. A pGKNeo cassette flanked by loxP sites was inserted into the gene as described in the schematic. The targeting construct can be linearized using Notl prior to electroporation into ES cells. The total size of the targeting construct (including vector backbone and Neo cassette) is ~17.2 kb.

PCR Screening of F1 Pups: PCR Screening Strategy Primer sets Al/N1 and A2/N1 were used to screen F1 pups. N1 anneals inside the Neo cassette and Al and A2 anneals 5′ to the short homology arm, outside the region used to create the targeting construct. Al/N1 amplifies a fragment that is 1.7 Kb in length, and A2/N1 amplifies a fragment that is 1.8KB in length. The expanded ES cell clones were used as a positive control. Oligos for PCR screening: Al: 5′-TAGCATGGAACTCATTCACAG -3′; A2: 5′-TCCTTAGATGCTAATTCTGTGG -3′; Ni: 5′-TGCGAGGCCAGAGGCCAGTTGTGTAGC-3′

Genotyping of pups was performed using the following primer sets: Primer set (I): detects the wild-type allele and the hypomorphic allele containing the full construct including exons 7, 8, and 9, NEO cassette, FRT and loxP sites (5′- ggc aga aac cca att aca tca a-3′; 5′ ggg ggc cca ggc aac atc t 3′); Primer set (II) detects the allele deleted of exons 7, 8, and 9 (5′ ggc aga aac cca att aca tca a 3′; 5′ cga ggc gtc cga aga ac 3′); Primer set (III) detects the wild-type allele and the allele deleted of exons 7, 8, and 9 (5′ gga ttt ggc tag gtt tgt tgg cag gtc a 3′; 5′ acg ggg cat ttg ggg ttc tcc 3′).

### Field Emission Scanning Electron Microscopy (FESEM)

Primary cultures of lung fibroblasts from Sec13^+/+^ and Sec13^H/−^ mice were prepared for FESEM imaging as previously described^47^,^48^. Briefly, cells were detached by trypsinization, washed in PBS, and subjected to two separate rounds of hypotonic treatment (15 mM Tris-HCl, pH 7.4, 10 mM NaCl, 3 mM MgCl_2_). The cells were then resuspended in PBS+10% glycerol, spun down onto poly-lysine-coated silicon chips and fixed in 3% glutaraldehyde in PBS. Further processing for FESEM included postfixation in 1% osmium tetroxide, dehydration through a graded ethanol series and critical-point drying on a CPD030 apparatus (Bal-Tec). Samples were sputter-coated with 1–2 nm chromium on an EMITECH K575X apparatus and imaged with an in-lens detector for secondary electrons on a Zeiss ULTRA plus field emission scanning electron microscope.

### Oligo-dT *in situ* Hybridization and Immunofluorescence Microscopy

Oligo-dT *in situ* hybridization was performed as we previously reported^49^. Immunofluorescence microscopy to detect hnRNP A1 (antibody was a gift from M. Matunis) and HDAC1 (monoclonal antibody 2E10 from Upstate Biotechnology) was performed as reported^50^ and Sec13 was detected as we previously described^12^. ER staining in lymphocytes from Sec13^+/+^ and Sec13^H/−^ mice was performed by plating these cells on coverslips treated with Cell-Tak (Corning). Cells were then fixed in 4% PFA for 15 min and permeabilized in 0.5% Triton X-100 for 4 min. Next, cells were blocked in 10% BSA for 30 min followed by incubation with 1 μg/ml anti-PDI antibody (Enzo Life Sciences). Cells were then incubated with anti-mouse Alexa Fluor 488 for 1h. Cells were washed 3x in PBS and then mounted in Fluoromount with Hoechst staining. Images were obtained with an Axiovert 200 100X objective, NA: 1.3., Deconvolved using AutoQuant software, and prepared using Imaris and ImageJ. Staining of ER exit sites and Golgi apparatus was performed in lung fibroblasts from Sec13^+/+^ and Sec13^H/−^ mice grown on glass coverslips. Cells were fixed and permeabilized for 15 min in methanol at −20 °C. Cells were then incubated with a mouse monoclonal antibody against p115 (4H1) and rabbit polyclonal antibodies against Sec31/p137 (Dr. F. Gorelick, Yale University School of Medicine, New Haven, CT)^51^ followed by goat anti-rabbit Alexa Fluor 488 and goat anti-mouse Alexa Fluor 594 (Invitrogen) secondary antibodies. DNA was stained with 1 μg/ml Hoechst 33342 (Invitrogen) for 5 min and coverslips were mounted on Mowiol 4–88 solution (Calbiochem). Epifluorescence images were acquired using an Axiovert 200M microscope (Zeiss), an LD Plan-Neofluar 40×/1.3 DIC objective (Zeiss), an Orca 285 camera (Hamamatsu) and the software Openlab 4.0.2 (Improvision).

### Immunohistochemistry

Liver and spleen were harvested from anesthetized wild-type and mutant mice following fixation via transcardial perfusion and overnight immersion in buffered 4% paraformaldehyde. Subsequent paraffin processing, embedding, and sectioning were performed by standard procedures^52^,^53^. Immunohistochemistry was then performed with mouse monoclonal anti-sera to protein disulfide-isomerase (PDI; ID3 clone, Enzo Life Sciences). Following deparaffinization, pH 6.0 citrate-based-microwave-antigen-retrieval, and blocking against detection of endogenous mouse IgG, serial sections were subjected to either PDI primary antibody (1 μg/ml) or phosphate buffered saline (no-primary substitution control). Bound primary antibody was detected according to previously described immunoperoxidase methods^54^,^55^. Review and photography of stained histologic preparations were carried out on a Leica DM2000 photomicroscope equipped with bright-field illumination. Photomicrography was achieved using this microscope and an Optronics Microfire digital CCD color camera interfaced with Macintosh G4 computer. Images were captured using PictureFrame 2.0 acquisition and software (Optronics, Inc.) and processed with Adobe Photoshop CS4.

### Transmission Electron Microscopy

Spleens from Sec13^+/+^ and Sec13^H/−^ mice were cut into 1 mm^3^ pieces and fixed with 2.5% (v/v) glutaraldehyde in 0.1 M sodium cacodylate buffer. Tissues were then rinsed in 0.1 M sodium cacodylate buffer and post-fixed in 1% osmium tetroxide and 0.8% Potassium Ferricyanide in 0.1 M sodium cacodylate buffer for 1.5 h at room temperature. Tissues were rinsed with water and en bloc stained with 4% uranyl acetate in 50% ethanol for 2 h. Next, tissues were dehydrated with increasing concentrations of ethanol, transitioned into resin with propylene oxide, infiltrated with Embed-812 resin, and polymerized in a 60 °C oven overnight. Blocks were sectioned with a diamond knife (Diatome) on a Leica Ultracut 6 ultramicrotome (Leica Microsystems) and collected onto copper grids, post-stained with 2% aqueous Uranyl acetate and lead citrate. Images were acquired on a Tecnai G^2^ spirit transmission electron microscope (FEI) equipped with a LaB_6_ source using a voltage of 120 kV.

### Real Time-RT PCR and Immunoblots

Real Time-RT PCR and western blots were performed as we previously described^56^,^57^. Briefly, cells were isolated from spleen and RNA was extracted by TRIZOL (Sigma). Reverse transcription was performed using the iScript cDNA synthesis kit (Biorad). Quantitative PCR was performed using LightCycler 480 SYBR Green I Mix (Roche) on the Roche 480 LightCycler Instrument. ΔΔCt values were calculated with the resulting Ct values compared to controls. Primers: β-actin (forward: 5′-TAGCACCATGAAGATCAAGAT-3′, reverse: 5′-CCGATCCACACAGAGTACTT-3′), IL-6 (forward: 5′-TCCATCCAGTTGCCTTCTTG-3′, reverse: 5′-GGTCTGTTGGGAGTGGTATC-3′), IL-10 (forward: 5′-TACTTGGGTTGCCAAGCCTTATCG-3′, reverse: 5′-TCTTCAGCTTCTCACCCAGGGAAT-3′), IFNγ (forward: 5′-TCAAGTGGCATAGATGTGGAAGAA-3′, reverse: 5′-TGGCTCTGCAGGATTTTCATG-3′) and TGF-β (forward: 5′-CCTGAGTGGCTGTCTTTTGA-3′, reverse: 5′-CGTGGAGTTTGTTATCTTTGCTG-3′). Nucleoporin antibodies were used as previously described^5^,^12^. Immunoblots were quantified by ImageJ software.

### Measurements of Albumin, Cholesterol and Bilirubin Levels

Serum levels of Albumin (ALB), Cholesterol (CHOL), and total bilirubin (TBIL) were run in the VITROS-25, using the VITROS ALB Slide method and VITROS Chemistry Systems.

### Cell Preparations

Spleen and mesenteric lymph nodes were removed and cell suspensions were prepared using a tissue homogenizer followed by centrifugation. Spleen cells were depleted from erythrocytes. Peritoneal macrophages were collected 3 days after injection of 2 ml 3% thioglycolate solution i.p. Lungs cells were obtained by digestion with collagenase type IV as previously described^58^.

### Immunization/Antigens

Mice were immunized subcutaneously (s. c.) at the base of the tail with 100 μg OVA emulsified in complete Freund’s adjuvant (CFA) containing 50 μg *Mycobacterium tuberculosis* H37RA (DIFCO). Hen’s egg ovalbumin (grade III) and Concanavalin A were purchased from Sigma. Fourteen days post-immunization, mice were sacrificed, and spleen were harvested for FACS and cell culture assays.

### Cytokine Assays

Cells isolated from spleen (erythrocyte depleted) were cultured at 1 × 10^7^ cells/ml with or without 4 mg/ml Concanavalin A (Con A). Supernatants were collected after 48 h, and cytokine levels were measured by capture ELISA. Plates were coated with purified monoclonal antibodies against specific cytokines (IL-6, IFN-γ and TGF-β) and cytokine binding was detected by horseradish-peroxidase (HRP)-labeled rat monoclonal antibodies purchased from BD Biosciences. For TGF-β measurements, specific antibodies were purchased from R & D. For intracellular staining, primary cultures were stimulated with Concanavalin A (ConA) for 12 h in the presence of 10 μg/ml brefeldin A from BD Bioscience. Cells were permeabilized using BD Cytofix/Cytoperm**®** Kits and then stained with phycoerithrin (PE)-conjugated mAbs from IL-10, IL-4, IFN-γ, IL-6 and IL-12. Flow cytometric analysis was performed on a FACSCalibur (BD Biosciences); at least 30,000 events were acquired for each sample and analysis was performed using FlowJo software (Tree Star Inc).

### Serum immunoglobulin assay (ELISA)

Total serum immunoglobulins (Ig), IgG and IgM as well as anti-OVA antibody titers were determined by enzyme linked immunosorbent assay (ELISA). Briefly, 96-well plates (Nunc, Roskild, Denmark) were coated overnight with either 0.1 μg/ml goat anti-mouse Ig (Southern Biotechnology Associates Birmingham, AL) for non-specific antibodies or 2 μg/ml OVA solution for anti-OVA antibodies in sodium carbonate buffer, pH 9.6, at 4 °C. Serum samples were added and, for antibody detection, plates were incubated with either horseradish-peroxidase(HRP)-labeled goat anti-mouse IgG1 or HRP-labeled goat anti-mouse Ig, IgM or IgG (Southern Biotechnology, Birmingham, AL) for 1 h at 37 °C. Plates were washed, and incubated in the dark with H_2_O_2_ in the presence of orthophenylenediamine (OPD, Sigma) in sodium citrate buffer, pH 5.0 for 20 min. Reaction was stopped with 20 μl of 2NH_2_SO_4_. Optical density was measured using an automatic ELISA reader at 492 nm. Results were calculated by running sum of ODs of serum dilutions between 1:100 and 1:12,800 of individual mice. This method represents a more precise measurement of antibody titers as described by our group^59^. Alternatively, the total Ig, IgM and IgG1 concentrations were obtained by interpolating a standard curve obtained by different concentrations of mouse polyclonal IgG and IgM antibodies (monoclonal OVA-14, Sigma).

### Analysis of Cell Markers and Intracellular Cytokines by Flow Cytometry

Fluorescein isothiocyanate-conjugated (FITC) mAbs; phycoerithrin (PE)-conjugated mAbs; PE-Cy5-conjugated mAbs against cellular markers (CD4, CD8, CD19, CD5, CD11b, CD11c, CD25, Foxp3, LAP, CD14, CD16, CD138, CD95, CD1d, B220, CD21, CD23, BAFF-R, MHCI, MHCII, IgG, IgM) and cytokines (IL-4, IL-10, IL-6, IL-12, IFN-γ) were purchased from BD Biosciences. Surface staining was performed according to standard procedures at a density of 0.5–1 × 10^6^ cells per 25 μl, and volumes were scaled up accordingly. Intracellular staining for cytokine expression was performed using cultured cells in the presence of Brefeldin A. Flow cytometric analysis was performed on a FACSCalibur (BD Biosciences); at least 30,000 events were acquired for each sample and analysis was performed using FlowJo software (Tree Star Inc).

### Statements on Animal Protocols

- All experimental methods involving animal studies were carried out in accordance with the approved guidelines of the National Institutes of Health (NIH).

- Our experimental protocols were approved by the Institutional Animal Care and Use Committee (IACUC) at University of Texas Southwestern Medical Center. Our Animal Protocol Number (APN) is 2008-0069.

## Additional Information

**How to cite this article**: Moreira, T. G. *et al.* Sec13 Regulates Expression of Specific Immune Factors Involved in Inflammation *In Vivo*. *Sci. Rep.* **5**, 17655; doi: 10.1038/srep17655 (2015).

## Supplementary Material

Supplementary Information

## References

[b1] Mor A., White M. A. & Fontoura B. M. Nuclear Trafficking in Health and Disease. Current Opinion in Cell Biology 28, 28–35 (2014). 24530809 10.1016/j.ceb.2014.01.007 PMC4061247

[b2] Yarbrough M. L., Mata M. A., Sakthivel R. & Fontoura B. M. Viral Subversion of Nucleocytoplasmic Trafficking. Traffic, doi: 10.1111/tra.12137 (2013). PMC3910510 24289861

[b3] Castello A., Izquierdo J. M., Welnowska E. & Carrasco L. RNA nuclear export is blocked by poliovirus 2A protease and is concomitant with nucleoporin cleavage. Journal of cell science 122, 3799–3809, doi: 10.1242/jcs.055988 (2009). 19789179

[b4] Enninga J., Levy D. E., Blobel G. & Fontoura B. M. Role of nucleoporin induction in releasing an mRNA nuclear export block. Science 295, 1523–1525 (2002). 11809937 10.1126/science.1067861

[b5] Chakraborty P. *et al.* Nucleoporin levels regulate cell cycle progression and phase-specific gene expression. Developmental cell 15, 657–667 (2008). 19000832 10.1016/j.devcel.2008.08.020 PMC2835575

[b6] Egecioglu D. & Brickner J. H. Gene positioning and expression. Current Opinion in Cell Biology 23, 338–345, doi: 10.1016/j.ceb.2011.01.001 (2011). 21292462 PMC3097288

[b7] Faria A. M. C. *et al.* The Nucleoporin Nup96 is Required for Proper Expression of Interferon-Regulated Proteins and Functions. Immunity 24, 295–304 (2006). 16546098 10.1016/j.immuni.2006.01.014

[b8] Light W. H. *et al.* A conserved role for human Nup98 in altering chromatin structure and promoting epigenetic transcriptional memory. PLoS biology 11, e1001524, doi: 10.1371/journal.pbio.1001524 (2013). 23555195 PMC3608542

[b9] Zhang Y. & Li X. A putative nucleoporin 96 Is required for both basal defense and constitutive resistance responses mediated by suppressor of npr1-1,constitutive 1. Plant Cell 17, 1306–1316, doi: 10.1105/tpc.104.029926 (2005). 15772285 PMC1088004

[b10] Roth C. & Wiermer M. Nucleoporins Nup160 and Seh1 are required for disease resistance in Arabidopsis. Plant Signal Behav 7, 1212–1214, doi: 10.4161/psb.21426 (2012). 22902705 PMC3493398

[b11] Wiermer M. *et al.* Putative members of the Arabidopsis Nup107-160 nuclear pore sub-complex contribute to pathogen defense. Plant J 70, 796–808, doi: 10.1111/j.1365-313X.2012.04928.x (2012). 22288649

[b12] Enninga J., Levay A. & Fontoura B. M. Sec13 shuttles between the nucleus and the cytoplasm and stably interacts with Nup96 at the nuclear pore complex. Molecular and cellular biology 23, 7271–7284 (2003). 14517296 10.1128/MCB.23.20.7271-7284.2003 PMC230331

[b13] Siniossoglou S. *et al.* A novel complex of nucleoporins, which includes Sec13p and a Sec13p homolog, is essential for normal nuclear pores. Cell 84, 265–275 (1996). 8565072 10.1016/s0092-8674(00)80981-2

[b14] Capelson M. *et al.* Chromatin-bound nuclear pore components regulate gene expression in higher eukaryotes. Cell 140, 372–383, doi: 10.1016/j.cell.2009.12.054 (2010). 20144761 PMC2821818

[b15] Barlowe C. *et al.* COPII: a membrane coat formed by Sec proteins that drive vesicle budding from the endoplasmic reticulum. Cell 77, 895–907 (1994). 8004676 10.1016/0092-8674(94)90138-4

[b16] Lord C., Ferro-Novick S. & Miller E. A. The highly conserved COPII coat complex sorts cargo from the endoplasmic reticulum and targets it to the golgi. Cold Spring Harb Perspect Biol 5, doi: 10.1101/cshperspect.a013367 (2013). PMC3552504 23378591

[b17] Laouar Y., Sutterwala F. S., Gorelik L. & Flavell R. A. Transforming growth factor-beta controls T helper type 1 cell development through regulation of natural killer cell interferon-gamma. Nat Immunol 6, 600–607, doi: 10.1038/ni1197 (2005). 15852008

[b18] Sanjabi S., Zenewicz L. A., Kamanaka M. & Flavell R. A. Anti-inflammatory and pro-inflammatory roles of TGF-beta, IL-10, and IL-22 in immunity and autoimmunity. Current opinion in pharmacology 9, 447–453, doi: 10.1016/j.coph.2009.04.008 (2009). 19481975 PMC2755239

[b19] Chen M. L., Yan B. S., Bando Y., Kuchroo V. K. & Weiner H. L. Latency-associated peptide identifies a novel CD4+CD25+ regulatory T cell subset with TGFbeta-mediated function and enhanced suppression of experimental autoimmune encephalomyelitis. J Immunol 180, 7327–7337 (2008). 18490732 10.4049/jimmunol.180.11.7327 PMC2771858

[b20] Koretzky G. A. Multiple roles of CD4 and CD8 in T cell activation. J Immunol 185, 2643–2644, doi: 10.4049/jimmunol.1090076 (2010). 20724729

[b21] Roes J., Choi B. K. & Cazac B. B. Redirection of B cell responsiveness by transforming growth factor beta receptor. Proceedings of the National Academy of Sciences of the United States of America 100, 7241–7246, doi: 10.1073/pnas.0731875100 (2003). 12773615 PMC165860

[b22] Shlomchik M. J. & Weisel F. Germinal center selection and the development of memory B and plasma cells. Immunol Rev 247, 52–63, doi: 10.1111/j.1600-065X.2012.01124.x (2012). 22500831

[b23] Harris D. P., Goodrich S., Mohrs K., Mohrs M. & Lund F. E. Cutting edge: the development of IL-4-producing B cells (B effector 2 cells) is controlled by IL-4, IL-4 receptor alpha, and Th2 cells. J Immunol 175, 7103–7107 (2005). 16301612 10.4049/jimmunol.175.11.7103

[b24] Rosser E. C. & Mauri C. Regulatory B Cells: Origin, Phenotype, and Function. Immunity 42, 607–612, doi: 10.1016/j.immuni.2015.04.005 (2015). 25902480

[b25] Kato H. *et al.* Length-dependent recognition of double-stranded ribonucleic acids by retinoic acid-inducible gene-I and melanoma differentiation-associated gene 5. J Exp Med 205, 1601–1610, doi: 10.1084/jem.20080091 (2008). 18591409 PMC2442638

[b26] David-Watine B., Israel A. & Kourilsky P. The regulation and expression of MHC class I genes. Immunology today 11, 286–292 (1990). 1698378 10.1016/0167-5699(90)90114-o

[b27] Hegde N. R., Chevalier M. S. & Johnson D. C. Viral inhibition of MHC class II antigen presentation. Trends in immunology 24, 278–285 (2003). 12738423 10.1016/s1471-4906(03)00099-1

[b28] Park I. K., Shultz L. D., Letterio J. J. & Gorham J. D. TGF-beta1 inhibits T-bet induction by IFN-gamma in murine CD4+ T cells through the protein tyrosine phosphatase Src homology region 2 domain-containing phosphatase-1. J Immunol 175, 5666–5674 (2005). 16237056 10.4049/jimmunol.175.9.5666

[b29] Prochazkova J., Pokorna K. & Holan V. IL-12 inhibits the TGF-beta-dependent T cell developmental programs and skews the TGF-beta-induced differentiation into a Th1-like direction. Immunobiology 217, 74–82, doi: 10.1016/j.imbio.2011.07.032 (2012). 21903294

[b30] Ulloa L., Doody J. & Massague J. Inhibition of transforming growth factor-beta/SMAD signalling by the interferon-gamma/STAT pathway. Nature 397, 710–713, doi: 10.1038/17826 (1999). 10067896

[b31] Yan X., Liu Z. & Chen Y. Regulation of TGF-beta signaling by Smad7. Acta biochimica et biophysica Sinica 41, 263–272 (2009). 19352540 10.1093/abbs/gmp018 PMC7110000

[b32] Higashi K. *et al.* Interferon-gamma interferes with transforming growth factor-beta signaling through direct interaction of YB-1 with Smad3. The Journal of biological chemistry 278, 43470–43479, doi: 10.1074/jbc.M302339200 (2003). 12917425

[b33] Ataie-Kachoie P., Pourgholami M. H., Richardson D. R. & Morris D. L. Gene of the month: Interleukin 6 (IL-6). Journal of clinical pathology 67, 932–937, doi: 10.1136/jclinpath-2014-202493 (2014). 25031389

[b34] Koncz G. & Hueber A. O. The Fas/CD95 Receptor Regulates the Death of Autoreactive B Cells and the Selection of Antigen-Specific B Cells. Frontiers in immunology 3, 207, doi: 10.3389/fimmu.2012.00207 (2012). 22848207 PMC3404404

[b35] Bras A., Martinez A. C. & Baixeras E. B cell receptor cross-linking prevents Fas-induced cell death by inactivating the IL-1 beta-converting enzyme protease and regulating Bcl-2/Bcl-x expression. J Immunol 159, 3168–3177 (1997). 9317114

[b36] Carter R. H. & Fearon D. T. CD19: lowering the threshold for antigen receptor stimulation of B lymphocytes. Science 256, 105–107 (1992). 1373518 10.1126/science.1373518

[b37] Catlett I. M. & Bishop G. A. Cutting edge: a novel mechanism for rescue of B cells from CD95/Fas-mediated apoptosis. J Immunol 163, 2378–2381 (1999). 10452970

[b38] Catlett I. M., Xie P., Hostager B. S. & Bishop G. A. Signaling through MHC class II molecules blocks CD95-induced apoptosis. J Immunol 166, 6019–6024 (2001). 11342618 10.4049/jimmunol.166.10.6019

[b39] Huck S., Jamin C., Youinou P. & Zouali M. High-density expression of CD95 on B cells and underrepresentation of the B-1 cell subset in human lupus. Journal of autoimmunity 11, 449–455, doi: 10.1006/jaut.1998.0226 (1998). 9802928

[b40] Katayama I. Clinical analysis of recurrent hypergammaglobulinemic purpura associated with Sjogren syndrome. The Journal of dermatology 22, 186–190 (1995). 7738274 10.1111/j.1346-8138.1995.tb03368.x

[b41] Schneeberger E., Citera G., Heredia M. & Maldonado Cocco J. Clinical significance of anti-Ro antibodies in rheumatoid arthritis. Clinical rheumatology 27, 517–519, doi: 10.1007/s10067-007-0812-x (2008). 18183345

[b42] Levine A. M. Monoclonal gammopathy associated with HIV infection. Clinical infectious diseases : an official publication of the Infectious Diseases Society of America 43, 1206–1208, doi: 10.1086/508358 (2006). 17029143

[b43] Shen X. & Tomaras G. D. Alterations of the B-cell response by HIV-1 replication. Current HIV/AIDS reports 8, 23–30, doi: 10.1007/s11904-010-0064-2 (2011). 21161615 PMC3638746

[b44] Haas A. *et al.* Systemic antibody responses to gut commensal bacteria during chronic HIV-1 infection. Gut 60, 1506–1519, doi: 10.1136/gut.2010.224774 (2011). 21515549

[b45] Gandhi R. *et al.* Cutting edge: human latency-associated peptide+ T cells: a novel regulatory T cell subset. J Immunol 184, 4620–4624, doi: 10.4049/jimmunol.0903329 (2010). 20368276 PMC2904991

[b46] Oida T. & Weiner H. L. Overexpression of TGF-ss 1 gene induces cell surface localized glucose-regulated protein 78-associated latency-associated peptide/TGF-ss. J Immunol 185, 3529–3535, doi: 10.4049/jimmunol.0904121 (2010). 20720212 PMC2997468

[b47] Fichtman B., Shaulov L. & Harel A. Imaging metazoan nuclear pore complexes by field emission scanning electron microscopy. Methods in cell biology 122, 41–58, doi: 10.1016/B978-0-12-417160-2.00002-3 (2014). 24857724

[b48] Shaulov L. & Harel A. Improved visualization of vertebrate nuclear pore complexes by field emission scanning electron microscopy. Structure 20, 407–413, doi: 10.1016/j.str.2012.01.022 (2012). 22405000

[b49] Chakraborty P., Satterly N. & Fontoura B. M. Nuclear export assays for poly(A) RNAs. Methods 39, 363–369 (2006). 16935004 10.1016/j.ymeth.2006.07.002

[b50] Cansizoglu A. E., Lee B. J., Zhang Z. C., Fontoura B. M. & Chook Y. M. Structure-based design of a pathway-specific nuclear import inhibitor. Nat Struct Mol Biol 14, 452–454 (2007). 17435768 10.1038/nsmb1229 PMC3437620

[b51] Shugrue C. A. *et al.* Identification of the putative mammalian orthologue of Sec31P, a component of the COPII coat. J Cell Sci 112 (**Pt 24**), 4547–4556 (1999). 10574704 10.1242/jcs.112.24.4547 PMC5567750

[b52] Shehan D. C. & Hrapchak B. B. Theory and Practice of Histotechnology, 2nd edition. Battele Press (1980).

[b53] Woods A. E. & Ellis R. C. Laboratory Histopathology, A Complete Reference. Churchill - Livingston Press (1996).

[b54] Borvak J. *et al.* Functional expression of the MHC class I-related receptor, FcRn, in endothelial cells of mice. International immunology 10, 1289–1298 (1998). 9786428 10.1093/intimm/10.9.1289

[b55] Cianga P., Medesan C., Richardson J. A., Ghetie V. & Ward E. S. Identification and function of neonatal Fc receptor in mammary gland of lactating mice. European journal of immunology 29, 2515–2523, doi: 10.1002/(SICI)1521-4141(199908)29:08<2515::AID-IMMU2515>3.0.CO;2-D (1999). 10458766

[b56] Tsai P. L. *et al.* Cellular RNA Binding Proteins NS1-BP and hnRNP K Regulate Influenza A Virus RNA Splicing. PLoS pathogens 9, e1003460, doi: 10.1371/journal.ppat.1003460 (2013). 23825951 PMC3694860

[b57] Yarbrough M. L. *et al.* Primate-specific miR-576-3p sets host defense signalling threshold. Nature communications 5, 4963, doi: 10.1038/ncomms5963 (2014). PMC4170571 25232931

[b58] Manicassamy B. *et al.* Analysis of *in vivo* dynamics of influenza virus infection in mice using a GFP reporter virus. Proceedings of the National Academy of Sciences of the United States of America 107, 11531–11536, doi: 10.1073/pnas.0914994107 (2010). 20534532 PMC2895123

[b59] Oliveira R. P., Santiago A. F., Ficker S. M., Gomes-Santos A. C. & Faria A. M. Antigen administration by continuous feeding enhances oral tolerance and leads to long-lasting effects. Journal of immunological methods, doi: 10.1016/j.jim.2015.02.005 (2015). 25707356

