# Supplementary material for: Sec13 Regulates Expression of Specific Immune Factors Involved in Inflammation In Vivo
Source: Sci Rep. 2015 Dec 3;5:17655. doi: 10.1038/srep17655 (PMC4668385; doi:10.1038/srep17655)
Supplement: Supplementary Information [file srep17655-s1.pdf]

Sec13 Regulates Expression of Specific Immune Factors Involved in Inflammation *In Vivo*

Thais G. Moreira[1,4,7,] Liang Zhang[1,7,8], Lihi Shaulov[5,9] , Amnon Harel[5], Sharon K.Kuss[1], [2]Jessica Williams, [2]John Shelton, Bandarigoda Somatilaka[1], Joachim Seemann[1], Jue Yang[1], Ramanavelan Sakthivel[1], Daniel R. Nussenzveig[3,6], Ana M.C. Faria[4*], and Beatriz M.A. Fontoura[1*]

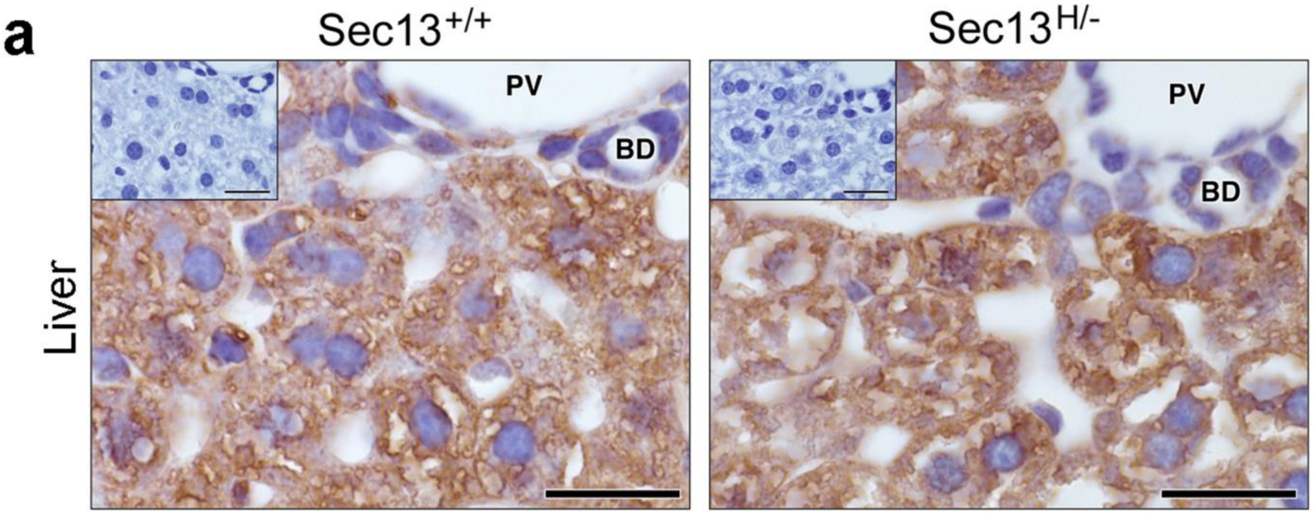

b

	Sec13 +/+	Sec13 H/-
**Albumin**	2.2g/dL ± 0.17	2.06g/dL ± 0.2
**Total Bilirubin**	0.63mg/dL ± 0.49	0.46mg/dL ± 0.46
**Cholesterol**	73mg/dL ± 5.29	58.33mg/dL ± 10.01

Supplementary Figure 1. ER Staining in the Liver and Serum Levels of Liver Factors from Sec13$^{+/+}$ and Sec13$^{H/-}$ mice. (**a**) Immunohistochemistry for protein disulfide-isomerase (PDI). High magnification bright-field microscopy of PDI immunoperoxidase localization in wild-type and mutant liver. Boundaries of hepatocyte endoplasmic reticulum are defined by PDI staining (brown) in the liver of wild-type and mutant mice. Hematoxylin counter stain is blue/purple and inset micrographs show absence of staining in matching-anatomy from adjacent sections not subjected to primary antibody. PV, hepatic portal vein; BD, bile duct; bars, 20µm. (**b**) Liver bulk secretory function was assessed by measuring serum levels of albumin, bilirubin, and cholesterol using the VITROS-25 system. The levels of these factors are not statistically different between Sec13$^{+/+}$ and Sec13$^{H/-}$ mice.

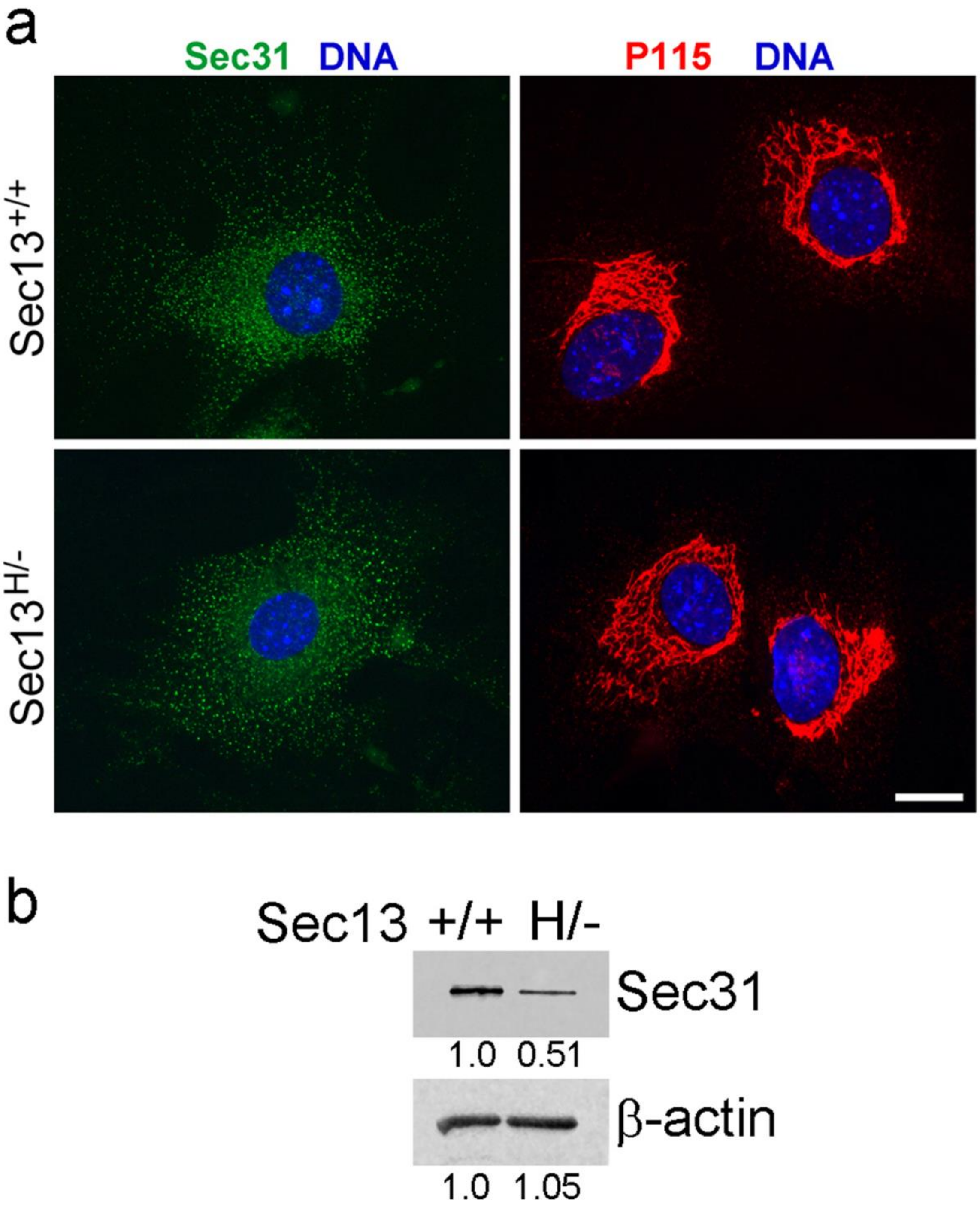

Supplementary Figure 2. Morphology of ER Exit Sites and Golgi from Sec13$^{+/+}$ and Sec13$^{H/-}$ mice. (**a**) Lung Fibroblasts from Sec13$^{+/+}$ and Sec13$^{H/-}$ mice were subjected to immunofluorescence microscopy for staining Sec31, to label ER exit sites (green), and the peripheral Golgi protein p115 (red). DNA is stained with Hoechst (blue). Bar, 20µm. (**b**) Immunoblot analysis of extracts from Sec13$^{+/+}$ and Sec13$^{H/-}$ lung fibroblasts was performed using antibodies against the depicted proteins. Immunoblots were quantified by ImageJ software.

Genotype	Cytokine	Treatment	Time (hours post-plating)	mRNA expression relative to wild-type (+/- standard deviation)	p value
Sec13$^{+/+}$	IL-6	-	0	1.00 ± 0.83	
Sec13$^{H/-}$	IL-6	-	0	0.60 ± 0.20	0.47
Sec13$^{+/+}$	IL-6	ConA	5	1.00 ± 0.54	
Sec13$^{H/-}$	IL-6	ConA	5	1.49 ± 0.24	0.22
Sec13$^{+/+}$	IL-6	ConA	24	1.00 ± 0.20	
Sec13$^{H/-}$	IL-6	ConA	24	0.80 ± 0.16	0.25
Sec13$^{+/+}$	IL-6	ConA	48	1.00 ± 0.20	
Sec13$^{H/-}$	IL-6	ConA	48	1.15 ± 0.27	0.47
Sec13$^{+/+}$	IL-6	-	48	1.00 ± 0.24	
Sec13$^{H/-}$	IL-6	-	48	1.77 ± 0.61	0.11
Sec13$^{+/+}$	IFN-γ	-	0	1.00 ± 1.13	
Sec13$^{H/-}$	IFN-γ	-	0	0.27 ± 0.07	0.33
Sec13$^{+/+}$	IFN-γ	ConA	5	1.00 ± 0.41	
Sec13$^{H/-}$	IFN-γ	ConA	5	1.08 ± 0.12	0.78
Sec13$^{+/+}$	IFN-γ	ConA	24	1.00 ± 0.36	
Sec13$^{H/-}$	IFN-γ	ConA	24	0.68 ± 0.09	0.21
Sec13$^{+/+}$	IFN-γ	ConA	48	1.71 ± 1.49	
Sec13$^{H/-}$	IFN-γ	ConA	48	1.36 ± 0.81	0.44
Sec13$^{+/+}$	IFN-γ	-	48	1.00 ± 0.53	
Sec13$^{H/-}$	IFN-γ	-	48	0.74 ± 0.26	0.15
Sec13$^{+/+}$	TGF-β	-	0	1.00 ± 0.47	
Sec13$^{H/-}$	TGF-β	-	0	1.67 ± 0.25	0.10
Sec13$^{+/+}$	TGF-β	ConA	5	1.00 ± 0.43	
Sec13$^{H/-}$	TGF-β	ConA	5	1.77* ± 0.24	0.05
Sec13$^{+/+}$	TGF-β	ConA	24	1.00 ± 1.12	
Sec13$^{H/-}$	TGF-β	ConA	24	1.21 ± 0.35	0.77
Sec13$^{+/+}$	TGF-β	ConA	48	1.00 ± 0.11	
Sec13$^{H/-}$	TGF-β	ConA	48	0.88 ± 0.07	0.02
Sec13$^{+/+}$	TGF-β	-	48	1.00 ± 0.09	
Sec13$^{H/-}$	TGF-β	-	48	0.91 ± 0.14	0.12
Sec13$^{+/+}$	IL-10	-	0	1.00 ± 0.80	
Sec13$^{H/-}$	IL-10	-	0	0.55 ± 0.19	0.40
Sec13$^{+/+}$	IL-10	ConA	5	1.00 ± 0.69	
Sec13$^{H/-}$	IL-10	ConA	5	1.37 ± 0.16	0.41
Sec13$^{+/+}$	IL-10	ConA	24	1.00 ± 0.18	
Sec13$^{H/-}$	IL-10	ConA	24	0.98 ± 0.11	0.87
Sec13$^{+/+}$	IL-10	ConA	48	1.00 ± 0.14	
Sec13$^{H/-}$	IL-10	ConA	48	0.99 ± 0.12	0.90
Sec13$^{+/+}$	IL-10	-	48	1.00 ± 0.16	
Sec13$^{H/-}$	IL-10	-	48	1.21 ± 0.24	0.28

Supplementary Figure 3. mRNA Levels of Cytokines from Sec13$^{+/+}$ and Sec13$^{H/-}$ mice. Cells were isolated from spleen of Sec13$^{+/+}$ or Sec13$^{H/-}$ mice and were unstimulated or stimulated with ConA. At the times indicated, real-time RT-PCR was performed for the cytokines denoted and results were normalized to β-actin. The Sec13$^{H/-}$ mRNA levels relative to the Sec13$^{+/+}$ mice per time point is displayed +/- standard deviation. Data represent at least 3 mice per group.

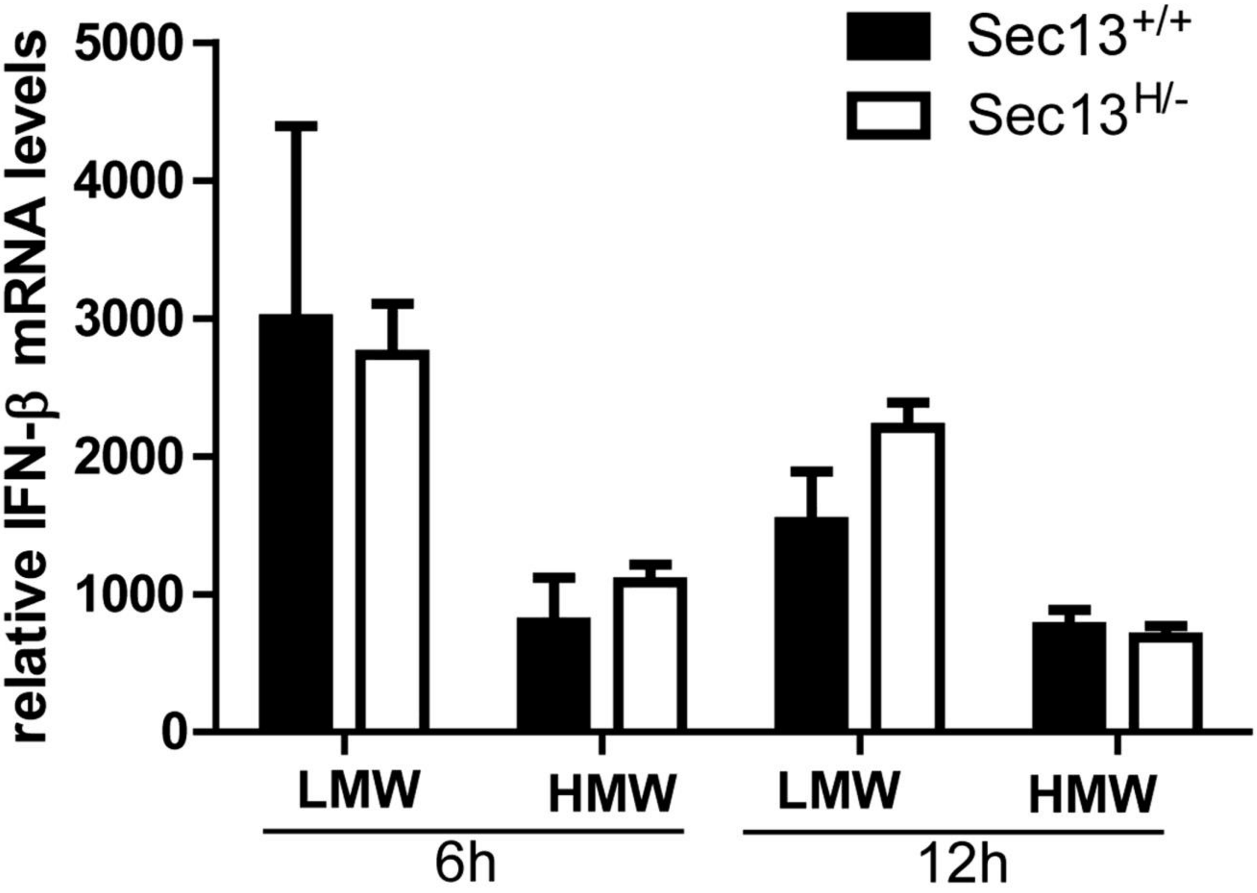

Supplementary Figure 4. Interferon-β Expression Is Not Altered in Sec13$^{H/-}$ mice. Lung fibroblasts from Sec13$^{+/+}$ or Sec13$^{H/-}$ mice were transfected with 1ug/ml of low molecular weight (LMW) or high molecular weight (HMW) poly (I:C) as indicated. After 6h, RNA was harvested and IFN-β mRNA levels were measured by RT-qPCR. The expression levels were normalized to β-actin.
